# Heterogeneous pathological outcomes after experimental pH1N1 influenza infection in ferrets correlate with viral replication and host immune responses in the lung

**DOI:** 10.1186/s13567-014-0085-8

**Published:** 2014-08-28

**Authors:** Beatriz Vidaña, Jorge Martínez, Pamela Martínez-Orellana, Lourdes García Migura, María Montoya, Jaime Martorell, Natàlia Majó

**Affiliations:** Departament de Sanitat i Anatomia Animals, Universitat Autònoma de Barcelona, Cerdanyola del Vallés, 08193 Bellaterra Spain; Departament de Medicina i Cirurgia Animals, Universitat Autònoma de Barcelona, Cerdanyola del Vallés, 08193 Bellaterra Spain; Centre de Recerca en Sanitat Animal (CReSA), UAB-IRTA, Campus de la Universitat Autònoma de Barcelona, Cerdanyola del Vallés, 08193 Bellaterra Spain; Institut de Recerca i Tecnologia Agroalimentaria (IRTA), Barcelona, Spain

## Abstract

The swine-origin pandemic (p) H1N1 influenza A virus causes mild upper-respiratory tract disease in most human patients. However, some patients developed severe lower-respiratory tract infections with fatal consequences, and the cause of these infections remain unknown. Recently, it has been suggested that different populations have different degrees of susceptibility to pH1N1 strains due to host genetic variations that are associated with inappropriate immune responses against viral genetic characteristics. Here, we tested whether the pathologic patterns of influenza strains that produce different disease outcomes in humans could be reproduced in a ferret model. Our results revealed that the severities of infection did not correspond to particular viral isolate and were not associated with the clinical phenotypes of the corresponding patients. Severe pathological outcomes were associated with higher viral replication, especially in alveolar areas, and with an exacerbated innate cellular immune response that was characterised by substantial phagocytic and cytotoxic cell migration into the lungs. Moreover, detrimental innate cellular responses were linked to the up-regulation of several proinflammatory cytokines and chemokines and the down-regulation of IFNα in the lungs. Additionally, severe lung lesions were associated with greater up-regulations of pro-apoptotic markers and higher levels of apoptotic neutrophils and macrophages. In conclusion, this study confirmed that the clinicopathological outcomes of pH1N1 infection in ferrets were not only due to viral replication abilities but also depended on the hosts’ capacities to mount efficient immune responses to control viral infection of the lung.

**Electronic supplementary material:**

The online version of this article (doi:10.1186/s13567-014-0085-8) contains supplementary material, which is available to authorized users.

## Introduction

In 2009, the swine-origin H1N1 influenza A virus (IAV) emerged and caused outbreaks of respiratory illness in humans around the world, and the World Health Organisation (WHO) declared a worldwide pandemic [1]. In contrast to seasonal influenza, which is more likely to cause severe illness in the elderly, children and immunocompromised patients, the pandemic H1N1 (pH1N1) caused serious disease in young adults and previously healthy individuals [2–4]. This phenomenon was related with the presence of cross-neutralising antibodies against pH1N1 in the elderly population but not in children and young adults [5].

The physiopathology of pH1N1 infection in humans differs across individuals. Whilst most patients developed mild upper respiratory-tract infection, some patients progressed to develop severe lower respiratory-tract complications with fatal consequences [4,6]. The cause of death in the severe cases was identified as a consequence of diffuse alveolar damage (DAD), which is also termed acute interstitial pneumonia [7].

The striking heterogeneity of the clinicopathological outcomes observed after pH1N1 infection in humans lead to numerous studies initially focused on the impact of viral evolution and mutation on the virulence of the infection. Virulence markers have been mapped to the polymerase genes (PB1, PB2 and PA), neuraminidases (NAs), and the non-structural proteins (NS1s) of the highly pathogenic avian influenza viruses and the 1918 pandemic H1N1 strains [8]. Recently, it has also been suggested that genetic polymorphisms that affect the polymerase complex and the hemagglutinin (HA) subunit may contribute to the pathogenicity of certain pH1N1 strains by conferring on them the ability to produce higher viral titres or the ability to replicate over a prolonged period of time [9,10]. The viral strains used in this work have been sequenced, and several mutations have been found in each of the viral strains [11]. However, the importance of these mutations in the pathogenicity of pH1N1 still needs to be clarified [11].

In contrast, several reports have suggested that influenza-associated pathology is most strongly determined by the different host factors [12–14]. Host genetic variation in immune-related genes has been shown to account for varying susceptibilities to numerous infectious agents and may contribute to the variation observed in pH1N1 susceptibility and disease severity [14–16]. A variety of studies have suggested that these host characteristics are associated with appropriate immune responses that may play important roles in determining the outcome of infection [13,17–19]. To date, polymorphisms in the chemokine receptor type (CCR) 5, toll like receptor (TLR) 3, tumour necrosis factor (TNF) and interferon-inducible transmembrane (IFITM) genes [15,20–22] and a deficiency in the immunoglobulin (Ig) G2 response [23,24] have been correlated with more severe courses of pH1N1 infection.

This study attempted to clarify whether the variance in virulence of pH1N1 isolates in humans significantly correlates with the clinicopathological outcome in an animal model of influenza infection. The aim of this study was accomplished by experimentally infecting ferrets with two pH1N1 isolates from two human patients who developed different clinical outcomes. The viral dynamics and host immune responses of the lungs of the infected ferrets were thoroughly investigated. Assessments of the pathologic patterns of the lungs and measurements of immune cell and cytokine responses and apoptotic cell induction were performed to identify the factors involved in the outcome of this disease.

## Material and methods

### Viruses

Two strains of human pH1N1 2009 IAV which had been isolated from geographically similar locations were used in the present study. The viruses were isolated at the National Influenza Centre, Centro Nacional de Microbiología, Instituto de Salud Carlos III (CNM, ISCIII) from respiratory samples sent by the Spanish Influenza Surveillance System for virological characterisation.

The pH1N1 2009 subtype isolates A/CastillaLaMancha/RR5661/2009 and A/CastillaLaMancha/RR5911/2009 were isolated from respiratory samples of two patients who were both Caucasian and free of previous co-morbid conditions at the time of infection. Hereafter, the following designations will be used: virus R61 for the A/CastillaLaMancha/RR5661/2009 virus, which was isolated from a 23-year old man exhibiting mild respiratory disease, and virus R11 for the A/CastillaLaMancha/RR5911/2009 virus, which was isolated from a 35-year old woman who developed a severe disease that resulted in a fatal outcome. Both viruses were isolated via bronchoalveolar lavage as described by Rodríguez et al. [11] and were passaged three times in Madin-Darby Canine kidney (MDCK) cells. The R61 virus had a titre of 10^8.3^ 50% Tissue Culture Infective Dose (TCID_50_) per mL, and the R11 virus had a titre of 10^8.2^ TCID_50_/mL as determined with the Reed and Muench method [25].

### Ferrets

Ten ferrets between the ages of twelve and twenty-four months that were seronegative for influenza A virus (influenza A antibody competition multi-species ELISA, ID Screen®, France) and Aleutian disease virus were randomly selected from a stable, purposely bred colony (Isoquimen, Spain). Upon arrival at the Centre de Recerca en Sanitat Animal (CReSA), the animals were placed in biosafety level 3 (BSL-3) facilities. The ferrets were randomly assigned to different experimental groups, separated into experimental isolation rooms and then maintained for one week for acclimation. The animals were maintained in standard housing cages and provided with commercial food pellets and tap water *ad libitum* throughout the experiment. All experiments were performed under a protocol (n° 1976) that was reviewed and approved by the Animal and Human Experimentation Ethics Commission of the Universitat Autònoma de Barcelona. All ferrets were monitored daily for clinical signs, and any animal that was determined to be in a moribund state was ethically euthanized. Any animal losing 25% of its original body weight or presenting severe respiratory signs such as severe tachypnea, nasal and conjunctival exudates and/or severe apathy (irresponsiveness to external stimulus) were considered to be in a moribund state and was humanely euthanized. Anorexia was measured daily by observing the amount of faeces and the amount of food that needed to be replaced and the attitude of the animals towards food. Depression was evaluated with reference to the activity of the animals and their response to external stimulus (such as sound or human presence). Tachypnea was considered when respiratory rate was higher than 70 breaths per minute, accompanied by abdominal breathing.

The animals were divided into three groups. The control group included two ferrets that were inoculated intratracheally with 0.1 M phosphate-buffered saline (pH = 7.5) (PBS). The R61 group included 4 ferrets that were infected with the R61 virus, and the R11 group included 4 ferrets that were infected with the R11 virus. The ferrets were intratracheally inoculated with 10^6^ TCID_50_/mL of the appropriate virus, and necropsies were performed on days 4 and 7 postinfection. The animals were euthanized by intravenous injection of sodium pentobarbital (100 mg/kg) under anaesthesia with ketamine (5–10 mg/kg) (Imalgene 1000® Merial, S.A., Spain) and medetomidine (0.05 mg/kg) (Domtor® Pfiser, S.A., Spain) and were then necropsied according to a standard protocol.

### Histopathology

Right lung lobe sections (cranial, medial and caudal), nasal turbinate and trachea were taken for histological examination according to standard protocols. The tissues were fixed for 48 h in neutral-buffered 10% formalin, then embedded in paraffin wax, sectioned at 3 μm, and stained with haematoxylin and eosin (HE) for examination by light microscopy.

Cross sections of the cranial, middle and caudal pulmonary lobes of each animal were separately evaluated, and semiquantitative assessments of IAV-associated microscopic lesions in the lungs were performed. The lesional scoring was graded on the basis of lesion severity as follows: grade 0 (no histopathological lesions observed), grade 1 (mild to moderate necrotising bronchiolitis), grade 2 (bronchointerstitial pneumonia characterised by necrotising bronchiolitis and diffuse alveolar damage in adjacent alveoli), and grade 3 (necrotising bronchiolitis and diffuse alveolar damage in the majority of the pulmonary parenchyma). Microscopic lesional scores were assigned for each lobe, and the means of the three lobes were used for the final histopathological score for each animal. Animals with lung lesion scores over 2 that presented with diffuse alveolar damage in at least 2 of the examined lung lobes were considered to have presented with severe lung lesions, and animals with scores below 2 were considered to have developed mild lung lesions.

### Viral detection and quantification

For detection of influenza A virus IAV antigen by immunohistochemistry (IHC), the tissues were stained with a primary antibody against the influenza A nucleoprotein (NP) as previously described [26]. Briefly, an antigen retrieval step was performed using protease XIV (Sigma-Aldrich, UDA) for 10 min at 37 °C and then blocked for 1 h with 2% bovine serum albumin (85040C, Sigma-Aldrich Química, S.A., Spain) at room temperature (RT). Samples were then incubated with a commercially available mouse-derived monoclonal antibody (ATCC, HB-65, H16L-10-4R5) concentration (343 mg/mL) as primary antibody at a dilution of 1:100 at 4 °C overnight followed by 1 h incubation with biotinylated goat antimouse IgG secondary antibody (Dako, immunoglobulins As, Denmark). Finally, an avidin-biotin-peroxidase complex (ABC) system (thermo Fisher Scientific, Rockjord, IL, USA) was used and the antigen –antibody reaction was visualized with 3.3′-diaminobenzidine tetrahydrochloride (DAB) such as chromogen. Sections were counterstained with Mayer’s Haematoxylin. Positive control consisted of formalin-fixed paraffin-embedded heart of chicken experimentally infected with influenza. The same sections in which the specific primary antibodies were substituted with PBS were used as negative controls. Semiquantitative assessments of influenza virus antigen expression in the lungs were performed, and the results were correlated with the lesional patterns. The positive cells in 6 arbitrarily chosen 20× objective fields in alveolar areas and 5 arbitrarily chosen 20× objective fields in bronchial or bronchiolar areas were quantified separately in each lung lobe (cranial, medial and caudal) for every animal. The mean of the total cell counts per field across the three lobes was calculated for each animal. The viral loads in the lung tissues were assessed by quantitative Reverse Transcription quantitative Polymerase Chain Reaction (RT-qPCR) following a Taq-Man one-step RT-qPCR performed with Fast 7500 equipment (Applied Biosystems, Foster city, CA, USA) as described previously [27]. Viral RNA was extracted with QIAamp viral mini kit (Qiagen, Valencia, CA, USA) obtaining 60 μL of eluted viral RNA. The matrix gene (M) fragment amplification was carried out using the primers showed in Table 1 and One-Step RT-PCR Master Mix Reagents (Applied Biosystems) following the manufacturer’s instructions using 5 μL of eluted RNA in a total volume of 25 μL. The amplification conditions were as follows: reverse transcription at 48 °C for 30 min; initial denaturation reaction at 95 °C for 15 min and 40 PCR-cycles of 95 °C 15 s and 60 °C 1 min. Standard curves and quantification were achieved by prior amplification of a 99 bp fragment of the M gene using One Step RT-PCR reagents (Qiagen) following manufacturer’s instructions. The M gene fragment amplicon obtained was cloned into pGEMT vector (Promega, Madison, WI, USA) and transformed by heat shock in *Escherichia coli* competent cells (Invitrogen, Paisley, UK). The recombinant plasmid was purified using the QIA prep Spin kit (Qiagen) and spectrophotometrically quantified (Qubit, Invitrogen) as described previously [28]. Serial 10-fold dilutions of both plasmids of known concentration were made and the standard curves were generated. The genome equivalent copies (GEC) of plasmid from the collected samples were determined based on these standard curves and by taking their volumes into account.

**Table 1:** **Ferret-specific gene primers with publication sources or database names and accession numbers**

**Gene**		**Primer Sequence (5′- 3′)**	**Publication source or NCBI accession n°**
pH1N1 (2009)	M + 25	F:AGATGAGTCTTCTAACCGAGGTCG R:TGCAAAGACACTTTCCAGTCTCTG	[27]
	M-124 human09		
	M + 64:	Probe:FAM^a^-TCAGGCCCCCTCAAAGCCGA-TAMRA^b^	
IFNα		F:TCTCCATGTGACAAACCAGAAGA	[31]
		R:CAGAAAGTCCTGAGCACAATTCC	
IFNγ		F: TCAAAGTGATGAATGATCTCTCACC	”
		R: GCCGGGAAACACACTGTGAC	
TNFα		F: CCAGATGGCCTCCAACTAATCA	”
		R: GGCTTGTCACTTGGAGTTCGA	
IL-6		F: AGTGGCTGAAACACGTAACAATTC	”
		R: ATGGCCCTCAGGCTGAACT	
CCL5		F: GCTGCTTTGCCTACATTTCC	”
		R: CCCATTTCTTCTGTGGGTTG	
CXCL8		F: AAGCAGGAAAACTGCCAAGAGA	”
		R: GCCAGAAGAAACCTGACCAAAG	
CXCL10		F: CTTTGAACCAAAGTGCTGTTCTTATC	”
		R: GCGTGTAGTTCTAGAGAGAGGTACTC	
TLR3		F: GATGACCTCCCAGCAAACAT	”
		R: GCACAATTCTGGCTCCAGTT	
CCL2		F:GCTCCCTATTCACTTGCTGTTTC	[55]
		R:GATTCGATAGCCCTCCAGCTT	
β-actin		F: GCAGGTCATCACCATCG	[Genbank: AF038150]
		R: TGGAGTTGAAGGTGGTCT	
IL-1α		F: GGGAAACTACCTCATGGC	[Genbank:JP011578]
		R: TAGAGTCACAGGAAATTTAGAATCTT	
CCL3		F: GGTCTTCTCTGCACCAT	[Genbank:JP007133]
		R: CCAGGCTTGGAGCATTG	
CASP8		F: TTATGACTTTAGCATAGCACGGA	[Genbank:JP006891]
		R: GTCTCTGAAAGGCACGAT	
BAX		F: CTGGACAGTAACATGGAGTTACA	[Genbank:JP006358]
		R: CAAAGTAGAAGAGGGCAACGA	

^a^FAM, 6-carboxylfluorescein.

^b^TAMRA, 6-carboxyltetramethyl rhodamine.

### Immunophenotyping and quantification of lung inflammatory cells

Phenotyping of the different inflammatory cell lineages following IHC were performed on the lungs of the infected and control animals to determine which inflammatory cell types were associated with the different lung lesional patterns. Neutrophils and macrophages were detected by IHC using anti-lysozyme polyclonal antibodies (Dako, Polyclonal Rabbit Anti-Human Lysozyme EC 3.2.1.17, ref A0099), T and B cells were detected using anti-CD3 (Dako, Polyclonal Rabbit Anti-Human CD3, n° IS503) and anti-CD20 (Thermo Scientific, CD20 Rabbit Polyclonal Antibody, ref RB-9013-P) polyclonal antibodies as previously described [29,30].

Cytotoxic lymphocytes and natural killer (NK) cells were detected using the anti-CD8a (Sino Biological, Polyclonal Rabbit anti-ferret CD8a Antibody, ref 60001-RPO2, China) polyclonal antibody. Briefly, tissue sections were deparaffinised with xylene and rehydrated through graded concentrations of alcohol. Endogenous peroxidase activity was blocked by incubation with 3% H_2_O_2_ in methanol for 30 min. Tissue sections were rinsed in PBS and immersed in Retrieval Solution (Dako, Target Retrieval solution 10x concentrate, n°S1699) for antigen retrieval. Later, the slides were blocked with 2% bovine serum albumin (85040C, Sigma-Aldrich Química, S.A., Spain) for one hour at RT and incubated with the primary antibody overnight at 4 °C at a dilution of 0.5 μg/mL. Next, a polymer-based non-avidin-biotin peroxidase system (Dako EnVision® + System, Peroxidase-HRP, Dako, Denmark) was applied directly to the slides and incubated for 30 min at RT. The reaction was developed with DAB (Sigma-Aldrich, Madrid, Spain) at RT, followed by counterstaining with Mayer’s haematoxylin. Ferret lymph node sections were used as positive controls. The same sections in which the specific primary antibodies were substituted with PBS were used as negative controls.

Semiquantitative assessments of the inflammatory cells detected in the lungs were also performed. The cells that were positive in the IHC for the anti-lysozyme (macrophages and neutrophils), anti-CD3 (T cells), anti-CD8a (cytotoxic T cells and NK cells) and anti-CD20 (B cells) antibodies were quantified for every animal and antibody. Positive cells in 6 arbitrarily chosen 40× objective fields in alveolar areas and 5 arbitrarily chosen 40× objective fields in bronchi or bronchiolar areas were counted separately for each lobe (cranial, medial and caudal) of every animal. The means of the total cell counts per field across the three lung lobes were then calculated for each animal and antibody.

### Cytokine and TLR gene expression profiles

The gene expressions of interferon (IFN) α, IFNγ, TNFα, interleukin (IL) 6, IL-1α, CXCL8, CCL5, CXCL10, CCL3, CCL2 and TLR3 were detected by RT-qPCR. Briefly, RNA extraction was performed on the ferret lung tissue samples with an RNeasy Mini Kit using the RNA stabilisation and on-column DNase digestion protocols (Qiagen). Reverse transcription was performed using an ImProm-II reverse transcription system (Promega) at 0.5 μg RNA. PCR was performed using a Power SYBR green kit (Applied Biosystems) and Fast 7500 equipment (Applied Biosystems). PCR reactions were performed in 10 μL reaction volumes using the Power SYBR green kit (Applied Biosystems); 40 amplification cycles were used, and the annealing temperature was 60°. The expression levels were normalised using the house-keeping gene β-actin, and the results are expressed as arbitrary units. The primer sequences and their publication sources are presented in Table 1. Primer sequences for the β-actin, IL-1α and CCL3 genes were designed as described previously [31]. The ferret-specific genes are available in the NCBI nucleotide database [32]. The designed primer sequences and the GenBank accession numbers are presented in Table 1. The amplification products were detected by electrophoresis to validate the sizes of the product in accordance with the primer design, and the products were purified using a QIAquick PCR Purification Kit (Qiagen). Sequencing reactions were performed with ABI Prism BigDye Terminator Cycle Sequencing v.3.1 Ready Reaction (Applied Biosystems) and analysed using an ABI PRISM model 3730 automated sequencer (Applied Biosystems). The amplified sequences correlated with the ferret specific target sequences.

### Apoptotic cell detection and quantification

The expression of the proapoptotic genes caspase 8 (CASP8) and BAX were quantified by RT-qPCR. The primer sequences are presented in Table 1. The RT-qPCR techniques were performed as previously described [31]. Briefly, RNA extraction was performed on the ferret lung tissue samples with an RNeasy Mini Kit using the RNA stabilisation and on-column DNase digestion protocols (Qiagen). Reverse transcription was performed using an ImProm-II reverse transcription system (Promega) at 0.5 μg RNA. PCR was performed using a Power SYBR green kit (Applied Biosystems) and Fast 7500 equipment (Applied Biosystems). PCR reactions were performed in 10 μL reaction volumes using the Power SYBR green kit (Applied Biosystems); 40 amplification cycles were used, and the annealing temperature was 60°. The expression levels were normalised using the house-keeping gene β-actin, and the results are expressed as arbitrary units. Primer sequences of CASP8 and BAX were designed as described previously [31]. The designed primer sequences and the GenBank accession numbers are presented in Table 1. Primer validation was performed as described above in the cytokine gene expression profile section.

Apoptotic cells in the lungs were also detected by IHC using the anti-caspase 3 polyclonal antibody (Cell Signalling, Cleaved Caspase-3 (Asp175) Antibody, ref 9661) diluted 1:100 in PBS using Ethylenediaminetetraacetic acid (EDTA) as the antigen retrieval method. The IHC technique was performed as described above for inflammatory cell immunophenotyping. Swine and ferret lymph node sections were used as positive controls, and the sections in which the specific primary antibodies were substituted with PBS were used as negative controls.

### Statistical analyses

Histopathological scores and positive cell quantification data were expressed as the means ± the standard errors of the mean (SEMs). The correlations between the IAV antigen quantifications and the histopathological scores of each animal were calculated by linear regression. Despite the small number of animals used in each infectious group, statistical analyses were performed. Comparisons of the immunophenotypes and the IAV antigen quantifications between the animals that were infected with the R11 and the R61 viruses and comparisons between animals with severe and mild lung lesions were performed using a non-parametric one-way analysis of variance (Kruskal-Wallis) followed when necessary by a Mann–Whitney *U* test. A robust analysis (one iteration) was used to obtain the means ± the SEMs for the RT-qPCR data. RT-qPCR comparisons between animals infected with the R11 and the R61 viruses and comparison between animals with severe and mild lung lesions were performed using Student’s *t*-tests. In all cases, the results were considered statistically significant when the *P* value was < 0.05. All statistical analyses and data visualisation were performed with GraphPad Prism 6 (GraphPad Software, La Jolla, CA, USA).

## Results

### Clinical signs

Two animals infected with the R61 virus and two animals infected with the R11 virus began to present with anorexia, depression and tachypnea at 2 days post infection (dpi).

Of these four animals, one animal infected with the R11 virus died at 4 dpi, and one animal infected with the R61 virus had to be sacrificed at 4 dpi for ethical reasons. Animals showing more severe clinical signs also presented higher body weight loss (data not shown) which also correlated with more severe pathological findings in the lungs. Temperature was not affected by viral group or by disease severity (data not shown).

### Gross lesions and histopathology

Gross pathological changes were observed mainly in the lungs of the infected animals that were necropsied at 4 dpi. One and two ferrets infected with the R11 and R61 viruses, respectively, presented with macroscopic lung lesions. The macroscopic lesions were characterised by heavy, firm, red and oedematous lungs. The animals in the control group did not exhibit any gross or histopathological changes.

**Figure 1:**
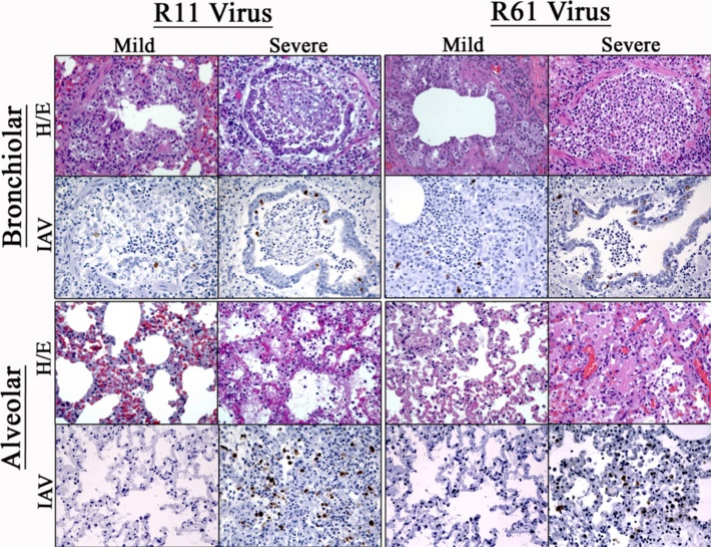
**Pulmonary histopathological lesions in pH1N1-infected ferrets at 4 dpi.** Pictures show different severity of lesions in alveolar and bronchiolar areas for R11 and R61 viruses. HE and immunohistochemical staining for IAV antigen (40x objective field).

The pulmonary histopathological scores are presented in Table 2. In general, the three lung lobes of any given animal were equally affected. No major differences in terms of histopathological scores were observed between the viral groups. Two animals infected with the R11 viral strain and two animals infected with R61 viral strain presented with severe lung lesions that were characterised by severe and diffuse alveolar damage. In general, the three lung lobes were equally affected, alveolar epithelial necrosis was observed, and the alveoli were distended and contained dense proteinaceous debris, desquamated cells, and high numbers of macrophages and neutrophils; in some cases, alveolar haemorrhage and hyaline membranes were also observed (Figure 1).

**Table 2:** **Characteristics of the experimental infection of ferrets with two different pH1N1 influenza viruses: viral inoculums, histopathological scores and pathological classifications**

**Animal**	**Sex**	**Viral inoculums**	**Days post infection**	**Histopathological score** ^**a**^	**Pathological classification**
**A**	Male	R11	4†	2.67 (0.47)	Severe
**B**	Male	R11	4	1 (0.00)	Mild
**C**	Female	R61	4	3 (0.00)	Severe
**D**	Female	R61	4	1 (0.00)	Mild
**E**	Male	R61	4	3 (0.00)	Severe
**F**	Male	R11	7	3 (0.00)	Severe
**G**	Male	R11	7	1.67 (0,94)	Mild
**H**	Male	R61	7	1 (0.00)	Mild
**I**	Female	PBS	Control	0 (0.00)	Control
**J**	Male	PBS	Control	0 (0.00)	Control

†Animal found dead.

^a^Mean and SEM of the histopathological scores of the three right lung lobes.

The remaining infected animals (two infected with the R11 virus and two infected with the R61 virus) presented with mild lung lesions that consisted of moderate to severe bronchial, bronchiolar and glandular necrosis with lymphoplasmacytic inflammation (Figure 1). The histopathological lesions observed at 7 dpi were similar to those described at 4 dpi but also included bronchiolar epithelial regeneration (data not shown).

The trachea and nasal turbinates were also microscopically examined. In general, the ferrets that presented with severe lung lesions also presented with necrotising rhinitis with mucous and tracheitis with a lymphoplasmacytic infiltration. The ferrets that presented with mild lesions also presented with lesions in the trachea and nasal turbinates that were characterised by a catarrhal rhinitis with mucous secretion and tracheal glandular necrosis (data not shown).

### Viral detection and quantification

IAV antigen expression was only detected in the lungs of the infected animals at 4 dpi, and no differences in IAV expression, as quantified by IHC (Additional file 1A) or RT-qPCR, were observed between animals in the R11 and R61 viral groups (Additional file 1B).

**Figure 2:**
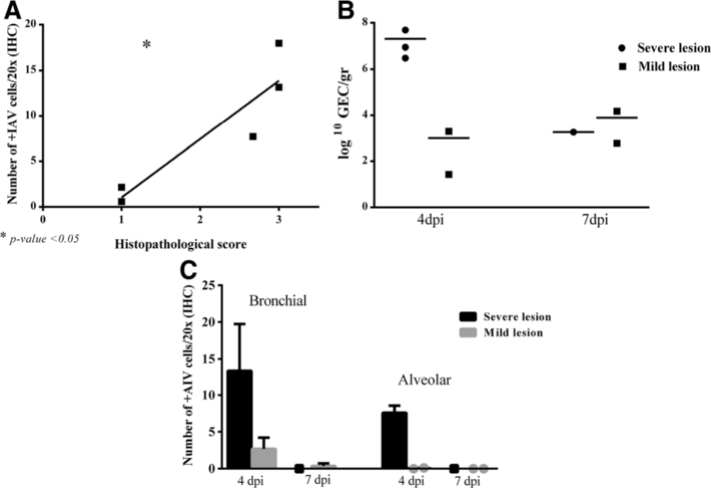
**Relation between histopathological score and quantification of viral antigen in pH1N1-infected ferrets. (A)** Linear regression shows the correlation between IAV antigen quantification and histopathological scores at 4 dpi, independently of viral strains (*p value < 0.05). **(B)** Relation between viral load measured by RT-PCR and histopathological score; GEC of plasmid per gram of lung tissue. **(C)** Immunohistochemical cell quantification of IAV-positive cells in ferret lungs segregated by histopathological score; Results express the mean cell counts with SEM (20× objective field).

Interestingly, IAV antigen quantifications in the lungs were significantly correlated with the histopathological scores of the infected animals (Figure 2A). Higher viral antigen quantifications and higher viral loads (Figure 2B and C) were observed in the lungs of the animals that presented with severe lung lesions than in those that presented with mild lung lesions at 4 dpi.

The animals that developed severe lung lesions exhibited viral antigen expression in bronchi/bronchiole epithelial cells and scattered expression in the alveolar parenchyma. Positive labelling was observed in the nuclei of type II pneumocytes and, to a lesser extent, in the type I pneumocyte nuclei and the nuclei and cytoplasm of macrophages and interstitial lymphocytic cells (Figure 1). In contrast, IAV antigen expression in the animals that developed mild lung lesions was restricted to the bronchi and bronchiolar areas (Figure 1), in which IAV antigens were mainly detected in the nuclei of the bronchi or bronchiolar epithelial and glandular cells.

### Immunophenotyping and quantification of lung inflammatory cells

**Figure 3:**
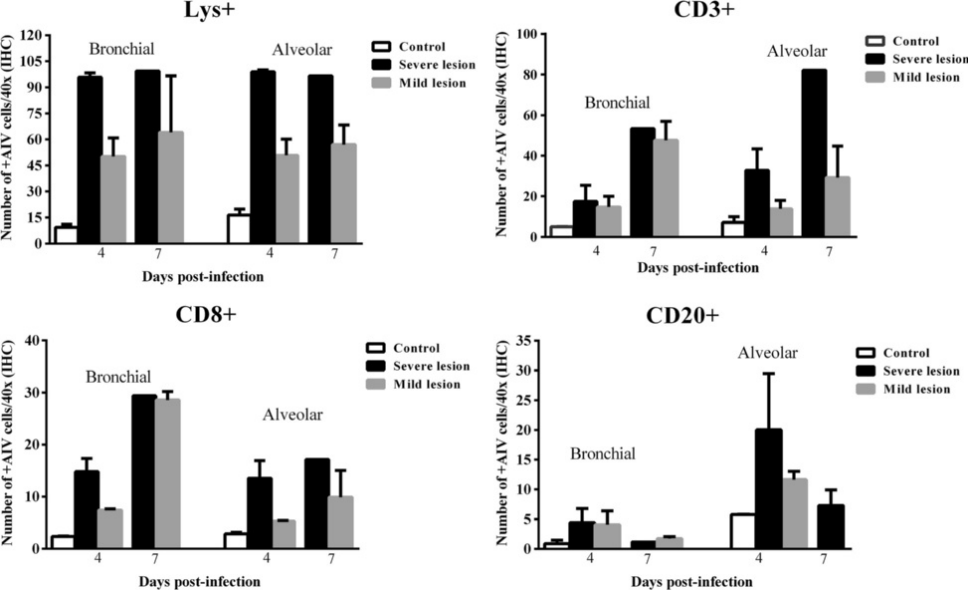
**Immunohistochemical quantification of inflammatory cells in ferret lungs infected with pH1N1 IAV.** Results express the mean cell counts with SEM (40× objective field) for lysozyme, CD3, CD8 and CD20-positive cells. Animals are segregated by histopathological score.

Quantifications of the different inflammatory cell populations for the animals grouped by histopathological score are illustrated in Figure 3.

**Figure 4:**
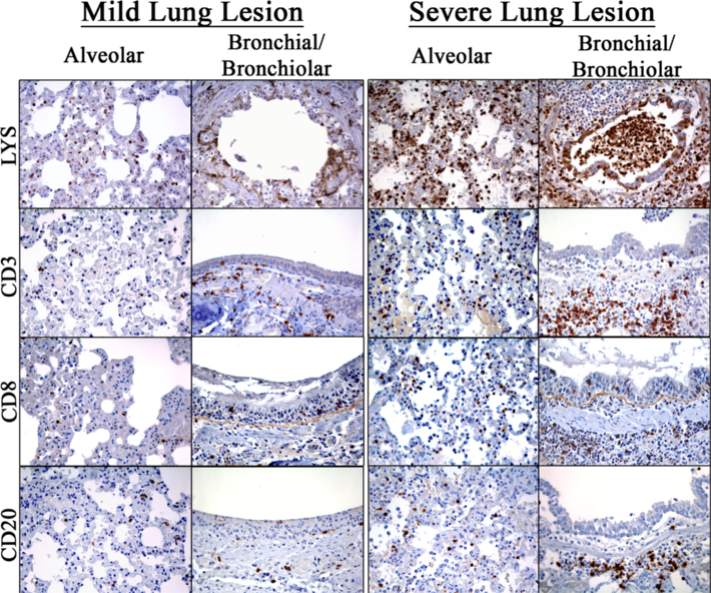
**Immunohistochemical caracterization of inflammatory cell populations in ferret lungs infected with pH1N1 IAV at 4 dpi.** IHC stain for positive cells. Haematoxylin counterstain (40× objective field).

Inflammatory cell averages were higher in the lungs of infected animals than in those of the control group. In general, lysozyme (Lys) positive cells were the most abundantly observed cells in the lungs of the infected animals, and the maximum cell counts were observed at 4 dpi. The lungs of the ferrets that presented with severe lung lesions exhibited elevations in lysozyme + cell averages in both the alveolar and the bronchi-bronchiolar compartments compared to the animals with mild lung lesions (Figure 3), particularly in the alveolar areas (Figure 4).

The CD3+ and CD8+ cell averages of the infected animals were higher at 7 dpi than at 4 dpi. More importantly, the CD3+ and CD8+ cell counts were higher in the ferrets that developed severe lung lesions (Figure 3), and this finding was particularly striking for the alveolar CD3+ cell averages (Figure 4). The CD8+ bronchial cell averages were similar in the animal that exhibited severe and mild lung lesions at 7 dpi.

In contrast to the observations regarding CD3+ and CD8+ cell quantifications, the CD20+ cell averages decreased in both histopathological groups at 7 dpi (Figure 3). In general, the CD20+ cell averages were the lowest among all of the inflammatory cells (Figure 4). The animals that presented with severe lung lesions exhibited slightly higher bronchial CD20+ cell counts at 4 dpi but not at 7 dpi (Figure 3).

### Cytokine and TLR gene expression profiles

**Figure 5:**
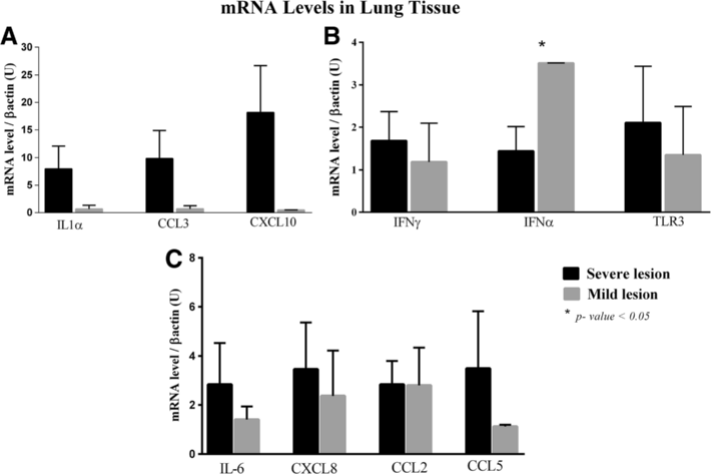
**RT-qPCR quantification of the genes expressed in pH1N1-infected ferrets with severe and mild lung lesions.** Comparisons of the cytokine, chemokine and TLR3 gene expression levels in lungs at 4 dpi. Gene expression of IL-1α, CCL3 and CXCL10 **(A)**; CXCL8, IL-6, CCL2 and CCL5 **(B)** and IFN-α, IFN-γ and TLR3 **(C)**. The data is presented as the means and the SEMs, (*p value < 0.05).

To determine the relationships between the observed immunophenotypes, the different pathological outcomes and the cytokine, chemokine and TLR gene expressions, RT-qPCR was performed. Both infected groups exhibited elevated gene expression of pro-inflammatory markers and TLR3 compared to the control group at both 4 and 7 dpi (data not shown). The gene expression profiles of the lungs of the animals that presented with severe lung lesions exhibited increased expressions of CXCL8, CCL3, IFNγ, CXCL10, IL-1α, CCL5, IL-6 and TLR3 compared to those of the animals that presented with mild lung lesions at both 4 dpi (Figure 5) and 7 dpi (data not shown). Interestingly, at 4 dpi, a statistically significant elevation in the induction of IFNα was observed in the lungs of the animals that presented with mild lung lesions (Figure 5B). No differences in CCL2 expression were observed between the pathological groups (Figure 5C). The expressions of all cytokines decreased in both pathological groups at 7 dpi with the exceptions of CCL2 and TLR3 in the animals with severe lung lesions (data not shown).

### Apoptotic cell detection and quantification

**Figure 6:**
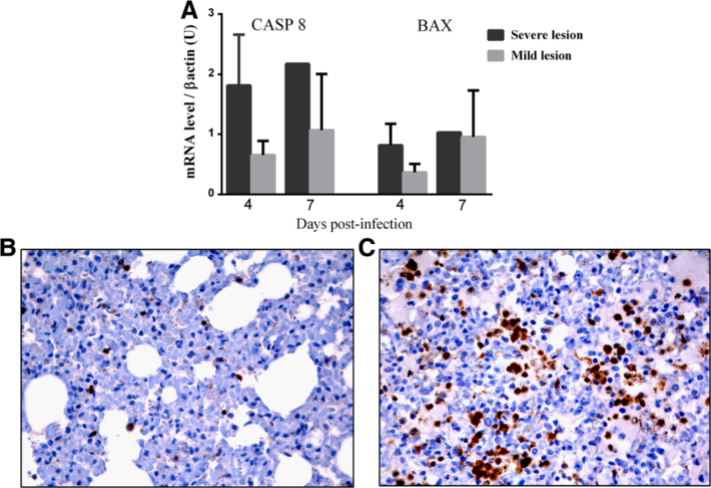
**Gene expressions of proapoptotic genes and IHC of apoptotic cells in lungs of pH1N1-infected ferrets at 4 and 7 dpi. (A)** Comparison of BAX and CASP8 gene expression levels in lungs with both severe and mild lesions as measured by RT-qPCR. The data expresses the means with SEMs. **(B)** and **(C)** IHC staining for caspase3 in the lungs of the ferrets with mild **(B)** and severe **(C)** lesions; haematoxylin counterstain (40× objective field).

The apoptotic genes BAX and CASP8 in the lungs of the infected animals were analysed with RT-qPCR. The gene expressions of BAX and CASP8 were elevated in both infected groups compared to the control group (data not shown). However, comparison of the apoptotic induction in the lungs of the animals that presented with different grades of lung pathology revealed that the animals with severe lung lesions exhibited elevations in the inductions of both apoptotic markers compared to the animals with mild lung lesions at both 4 and 7 dpi (Figure 6A).

To determine which cells were involved in the increases in pro-apoptotic gene expression in the animals with severe lung lesions, IHC using the anti-caspase 3 antibody was performed. As expected, the numbers of apoptotic cells were elevated in the ferrets with severe lung lesions compared to the animals with mild lesions, but the positive cells were mainly consistent with macrophages and neutrophils (Figure 6B and C), which accords with the phagocytic cell averages observed in each pathological group.

## Discussion

The pathogenic features of severe IAV infection result from complex and dynamic processes that involve various components of the host immune system and their responses to virus-induced changes. Understanding both virus and host response characteristics in individuals who develop mild or severe disease is important for the future development of therapeutic strategies for cases of severe influenza infection.

In this study, ferrets were infected with two pH1N1 isolates from two patients who developed significantly different clinical outcomes. Although the number of animals used in each infectious group is limited, our results demonstrated that the virulences of the pH1N1 strains in ferrets were not associated with the clinical phenotypes of the corresponding human patients. Similar findings have been described by other studies that have used animal models that have been infected with pH1N1 isolates from patients who exhibited diverse degrees of disease severity [9,33]. Indeed, the severity of infection in ferrets can range from a sublethal infection with mild symptoms to lethality rates of 30-50% [5,34,35]; this variability suggests the importance of the influences of both differences in viral strains and individual host variability on the severity of infection.

A previous study that employed the viral isolates used in this trial showed differences in the in vitro and in vivo characteristics of the two strains. In that study, the R11 strain exhibited higher in vitro replication rates between 9 and 24 hours post infection and 2 dpi in mice, and this difference was accompanied by more severe pathological lesions relative to those induced by the R61 strain [11]. As previously stated, in this study, the virulence of the R11 isolate was not consistently observed in ferrets, and more surprisingly, the R61 strain induced unexpectedly high viral titres in the lungs that were associated with severe pathological lesions in two animals. These contradictory results may be attributable to the following explanations: i) the mouse model may not be the most suitable model for assessing influenza virus pathogenicity [36–38], and ii) ferrets, as an outbreed animal, may exhibit differential individual susceptibilities to viral infection. In humans, the genetic factors of the host are increasingly being linked to disease courses [12,13]. Host-specific characteristics, such as the presence of homozygous CCR5 deleterious alleles (*CCR5*_*D*_*32)*, have been found to be associated with poor outcomes of pH1N1 infection [14–16,20]. Interestingly, the patient from which the R11 virus was isolated was recently found to have this genotype [11].

Our results revealed a significant correlation between lung lesional score and virus quantification in the lung that was independent of the viral strain that was inoculated. This finding led us to further investigate the immune responses and viral kinetic patterns by grouping the animals according to lung lesion severity rather than only considering the infecting viral strain.

Viral quantifications and localisations of the viral antigens in the lung compartments differed between individuals that were infected with the same virus but correlated with the severity of the lung lesions. Interestingly, in the animals that developed severe lung lesions, the viral antigen was observed in the alveolar areas, which suggests that sustained viral replication in the alveolar parenchyma was critical for the establishment of severe pathology; this suggestion has previously been made by Kuiken et al. [39]. In contrast, the animals that developed mild lung lesions did not present with IAV antigens in the alveolar areas and their viral quantifications were significantly lower.

Although viral replication is fundamental in determining the severity of the disease, the inflammatory response to the presence of the virus also plays important roles in tissue damage and pathological outcomes [40]. The induction of proinflammatory cytokines and chemokines after influenza infection is known to promote respiratory tract inflammation by recruiting inflammatory cells to the site of infection [41]. In this work, we found that the ferrets that developed severe lung lesions exhibited elevated expressions of proinflammatory cytokines (IL-1α, IL-6 and IFNγ) and chemoattractants (CXCL8, CXCL10, CCL3 and CCL5), which correlated with increased levels of phagocytic and T cell infiltrates in the lungs. The most abundant cell populations were the Lys + phagocytic cells (neutrophils and macrophages) followed by the CD3+ and CD8+ T cell populations. The exacerbated recruitment of neutrophils and macrophages into the lung parenchyma and alveolar spaces is recognised as contributing to the detrimental effects of influenza immunopathology via the production of pro-inflammatory molecules and the release of reactive oxygen species and nitric oxide [41–44].

In the present study, CD3+ and CD8+ cells were found more abundantly in the ferrets that exhibited severe lesional pattern that were accompanied with elevations in the inductions of IFNγ, CCL5 and CCL3. Several studies have shown that NK and CD8 T cells concentrations increase in mouse and human lungs following severe influenza infection both in the early stages of influenza infection and the later viral clearance stage [41,45–49]. Moreover, the suppression of the early innate cell infiltration of NK and CD8 T cells has been shown to significantly increase mouse survival rates without altering the kinetics of viral clearance; these findings suggest that the infiltration of these cells has a negative inflammatory role that is independent of viral kinetics [50,51].

Several studies have identified common cytokine gene expression profiles that are involved in neutrophil recruitment, stimulation of T cell proliferation and CD8+ cell-mediated inflammation in influenza- associated pathology [17,52,53]. Gene expression profiles that promote T helper (Th) 1 cell responses have also been shown to be closely related to poor outcomes in severe cases of pH1N1 infection [17,54,55]. Here, the animals that developed severe lung lesions also presented with elevated expressions of Th1 response-inducers (CCL5, CCL3, CXCL10 and IFNγ), while no differences in the expression of the Th2 response-inducer CCL2 were observed between animals with severe and mild lesions.

IFNα gene expression in the animals that developed mild lung lesions was significantly elevated compared to that of the animals with severe lung lesions at 4 dpi; this finding suggests that IFNα has a protective role in the early stages of infection. Supporting this supposition, Svitek et al. found that ferrets that develop mild seasonal influenza also exhibit increases in the expression of IFNα [56]. Moreover, some studies of mice and macaques have shown that the exogenous administration of IFNα protects animals against influenza A and SARS coronavirus infections [57,58].

To determine the differences in viral signalling in the animals with different pathological outcomes, TLR3 gene expression was measured. TLR3 is an innate immune recognition receptor that has key roles in dsRNA detection, the initiation of innate immune responses, and the linking of innate and adaptive immunity to limit virus production [59]. It is known that aberrant TLR3 signalling plays a key role in mediating lung pathology and contributes to detrimental host inflammatory responses through the induction of proinflammatory molecules and the recruitment of CD8+ and phagocytic cells during IAV virus infection [42,47,59–61]. Polymorphisms that alter the function of TLR3 have been shown to be related to severe cases of pH1N1 and H5N1 influenza infection [20,60,62]. In the present work, the animals that developed severe lung lesions exhibited elevated TLR3 gene expression in the lungs that correlated with increased viral quantifications and increased recruitment of CD8+ and Lys + cells in these animals. However, the severely ill animals exhibited significantly lower expressions of IFNα, which is up-regulated by TLR3 induction. Nevertheless, this striking phenomenon may be indicative of inappropriate functioning of either the TLR3 or the Interferon receptor (IFNR)1 or impairments of the TLR3-IFNR1 pathway.

Yang et al. [63] have recently demonstrated that pH1N1 can induce caspase-3-dependent apoptosis in alveolar epithelial cells (AEC). In our study, the animals with severe lung lesions presented with increased inductions of both the BAX and CASP8 pro-apoptotic genes in the lungs. However, immunohistopathological analyses revealed that apoptotic cells at 4 and 7 dpi were mainly identified as macrophages and neutrophils. This finding may elucidate another mechanism of lung tissue damage that is mediated by a delayed clearance of inflammatory cells from infected lungs. The occurrence of complete resolution of inflammation requires apoptotic neutrophils to be cleared by local tissue macrophages. However, if either neutrophil apoptosis or the clearance of apoptotic neutrophils by macrophages is delayed, inflammation-related tissue damage occurs and leads to the exacerbation of inflammation [64,65] due to increased numbers of inflammatory cells in apoptosis and increased gene expression of BAX and CASP8 that were observed in the ferrets with severe lung lesions. Moreover, these findings are likely related to the delayed viral clearance and the positive feedback loop of proinflammatory cytokines.

This study represents an exhaustive characterisation of the inflammatory cell populations and proinflammatory gene expression profiles in the lungs of pH1N1-infected ferrets. Additionally, despite the limited number of animals used, we provided further evidence regarding the implications of viral replication efficiency, inflammatory cytokine induction, and phagocytic and cytotoxic cell infiltration in influenza-infected lungs that are associated with pathology at the time of the switch from the innate to the adaptive immune response. More importantly, our results support the notion that viral replication and the correct orchestration of the early inflammatory response are key factors in the development of severe influenza.

## Additional file

Additional file 1:
**Pulmonary IAV antigen quantification and viral load in ferrets infected with R11 and R61 pH1N1 viruses.** (A) Immunohistochemical quantification; results express the mean cell counts with SEM (20x objective field). (B) Viral load is measured by RT-qPCR. GEC of plasmid per gram of lung tissue.

## Abbreviations

ABC: Avidin-biotin-peroxidase complex
AEC: Alveolar epithelial cells
BSL-3: Biosafety level 3
CASP8: Caspase 8
CNM: Centro Nacional de Microbiología
CReSA: Centre de Recerca en Sanitat Animal
CCR: Chemokine receptor type
DAB: 3.3′-diaminobenzidine tetrahydrochloride
DAD: Diffuse alveolar damage
dpi: days post infection
EDTA: Ethylenediaminetetraacetic acid
GEC: Genome equivalent copies
HA: Hemagglutinin
HE: Haematoxylin and eosin
IAV: Influenza A virus
IFITM: Interferon-inducible transmembrane
IFN: Interferon
IFNR: Interferon receptor
Ig: Immunoglobulin
IHC: Immunohistochemistry
IL: Interleukin
ISCIII: Instituto de Salud Carlos III
Lys: Lysozyme
M: Matrix
MDCK: Madin-Darby Canine Kidney
min: Minutes
NAs: Neuraminidases
NK: Natural Killer
NP: Nucleoprotein
NS1s: Non-structural 1 protein
PBS: Phosphate-buffered saline
pH1N1: Pandemic H1N1
RT: Room temperature
RT-qPCR: Reverse transcription quantitative polymerase chain reaction
s: Seconds
SEMs: Standard errors of the mean
TCID_50_: 50% tissue culture infective dose
Th: T helper
TLR: Toll-like receptor
TNF: Tumor necrosis factor
WHO: World Health Organisation
