# Supplementary figures and images for: Heterogeneous pathological outcomes after experimental pH1N1 influenza infection in ferrets correlate with viral replication and host immune responses in the lung
Source: Vet Res. 2014 Aug 28;45(1):85. doi: 10.1186/s13567-014-0085-8 (PMC4161856; doi:10.1186/s13567-014-0085-8)

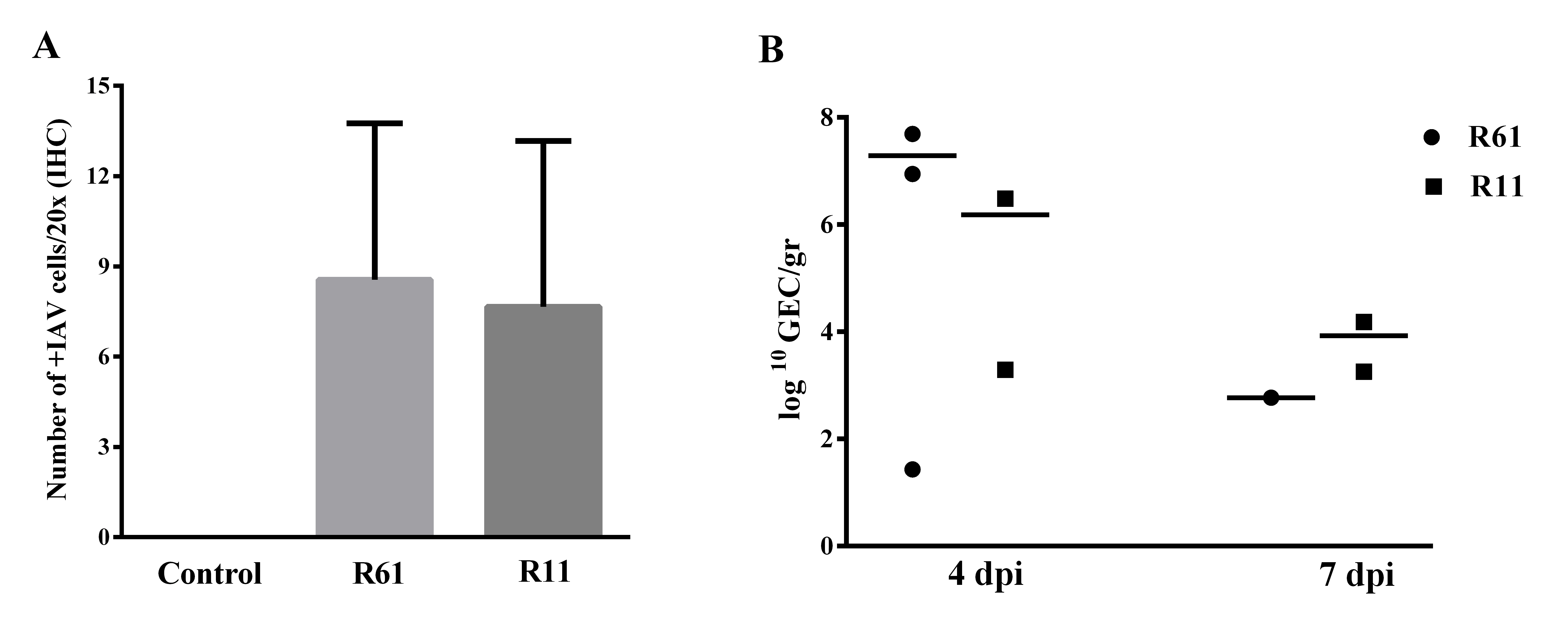

Supplement: Additional file 1: — Pulmonary IAV antigen quantification and viral load in ferrets infected with R11 and R61 pH1N1 viruses. (A) Immunohistochemical quantification; results express the mean cell counts with SEM (20x objective field). (B) Viral load is measured by RT-qPCR. GEC of plasmid per gram of lung tissue. [file 13567_2014_85_MOESM1_ESM.jpeg]
